# Video-Based System for Automatic Measurement of Barbell Velocity in Back Squat

**DOI:** 10.3390/s21030925

**Published:** 2021-01-30

**Authors:** Basilio Pueo, Jose J. Lopez, Jose M. Mossi, Adrian Colomer, Jose M. Jimenez-Olmedo

**Affiliations:** 1 University Institute for Computing Research, University of Alicante, 03690 Alicante, Spain; 2 Institute of Telecommunications and Multimedia Applications, Universitat Politècnica de València, 46022 Valencia, Spain; jjlopez@dcom.upv.es (J.J.L.); jmmossi@dcom.upv.es (J.M.M.); 3 Institute for Research and Innovation in Bioengineering, Universitat Politècnica de València, 46022 Valencia, Spain; adcogra@i3b.upv.es; 4 Physical Education and Sport, University of Alicante, 03690 Alicante, Spain; j.olmedo@ua.es

**Keywords:** smartphone, image processing, training, sports, validity, reliability, instrument, algorithm, homography

## Abstract

Velocity-based training is a contemporary method used by sports coaches to prescribe the optimal loading based on the velocity of movement of a load lifted. The most employed and accurate instruments to monitor velocity are linear position transducers. Alternatively, smartphone apps compute mean velocity after each execution by manual on-screen digitizing, introducing human error. In this paper, a video-based instrument delivering unattended, real-time measures of barbell velocity with a smartphone high-speed camera has been developed. A custom image-processing algorithm allows for the detection of reference points of a multipower machine to autocalibrate and automatically track barbell markers to give real-time kinematic-derived parameters. Validity and reliability were studied by comparing the simultaneous measurement of 160 repetitions of back squat lifts executed by 20 athletes with the proposed instrument and a validated linear position transducer, used as a criterion. The video system produced practically identical range, velocity, force, and power outcomes to the criterion with low and proportional systematic bias and random errors. Our results suggest that the developed video system is a valid, reliable, and trustworthy instrument for measuring velocity and derived variables accurately with practical implications for use by coaches and practitioners.

## 1. Introduction

Data collection is of paramount importance for sports scientists, coaches, athletes, and practitioners to objectively assess performance improvements. The measurement of any physical performance indicator gives valuable information to accurately prescribe individual-specific training, monitor changes, and supervise expected outcomes in practice and competition [1,2].

One of the most important physical performance metrics is the force exerted by an athlete during physical exercise, as adequate muscular strength is a basic capacity required to perform general sports skills and avoid injuries [3]. In recent years, the inverse relationship between force and velocity, such as the physical capacity to perform movements in the shortest time possible, has led to an optimized training method known as velocity-based training (VBT) [4,5,6]. Contrary to classical strength training in which the weights are pre-selected, based on the percentage value of maximum capacity, in VBT, the weights are dictated by the velocity that the athlete is attempting to train. Therefore, the velocity of movement must be monitored in real time to adjust the weight appropriately [7].

The velocity output can be measured by different technologies. First, linear position transducers (LPTs), which are composed of a string attached to a barbell and connected to a rotational encoder, have been used for decades as the gold standard because of the direct measurement of linear displacement, high sampling rate capture (around 1000 Hz), and reliability [8,9]. LPTs are expensive equipment with moving rotating parts and delicate strings that need appropriate use and limit the range of motion lifts [10,11]. Alternatively, inertial measurement units (IMUs) are able to record changes in gravitational acceleration in selected body parts (usually forearms) or barbells [12,13]. Although being very popular due to their cost and ease of use, the velocity and displacement values are estimated through time integration, which results in large errors for slow movements [13,14]. Velocity can also be assessed through an optoelectronic infrared camera, tracking the target with reflective markers [15]. Being a type of one-plane motion capture, this instrument requires the placement of markers and accurate calibration to obtain useful results. Finally, smartphone applications have recently emerged, allowing for manual video digitizing of barbell displacements to obtain velocity metrics [10,16,17]. Smartphone apps are affordable instruments used widely as alternatives to laboratory-based instruments. The capture precision of video-based instruments is a function of spatial (number of pixels) and temporal (frames per second) resolutions. However, the accuracy of such smartphone apps depends on the ability of the user to select specific video frames of the barbell displacement range once the movement is finished. Moreover, real-time feedback is of utmost importance in velocity-based training to monitor the acquired target. These apps cannot give instantaneous information of each repetition velocity as the video frames are selected manually after the completion of the exercise.

Barbell detection and tracking can also be performed by converting moving images into digital information using image processing methods. Using the projective geometry and homogeneous coordinates of the pinhole camera model, a mathematical framework can be derived to convert 3D to 2D imaging [18] and, with some length references in the scene, measurements of displacement and velocity can be done. For a system to be practical, it must be easy to set up and configure, work unattended as much as possible, perform fast enough to give real-time feedback at low cost. To achieve the above technical requirements in uncontrolled environments like gym rooms, automatic barbell detection with only one camera working in the visible spectrum is a very challenging task for image processing since depth information cannot be measured directly. Furthermore, the video technology of current smartphones allows for detection and tracking of moving parts of the image and computing corrections to pixel coordinates using homography transformation [19], thus reducing the cost of the instrument.

However, to the knowledge of the authors, there is no available instrument to measure weight velocity in VBT training by tracking barbell displacement using image processing with one camera in the visible spectrum. The only current approach in a similar app uses sophisticated technology (laser imaging detection and ranging) in very few expensive smartphone models [20]. Given the current computing processing power and high-speed camera systems, our method would allow the user to monitor the performance in real time to check that the target velocity is achieved without mechanical limitations.

In this paper, a video-based instrument that automatically assesses the barbell velocity using image processing is presented. The developed methods track barbell markers, analyze the kinematics of the movement, and give real-time VBT performance metrics to the user in a contactless and unattended way, avoiding errors due to human observation. To that end, two strips of available reference markers are attached to a multipower machine to compute actual dimensions using homography. This information is used to measure the displacements of a simple colored mark on the barbell in an unattended way. To check the viability of the proposal for current portable devices, video recordings were performed with a typical high-speed camera smartphone model. The instrument was tested by comparing a set of real executions with a validated LPT serving as the gold standard.

## 2. Materials and Methods

### 2.1. Experiment Procedure Setup

**Figure 1:**
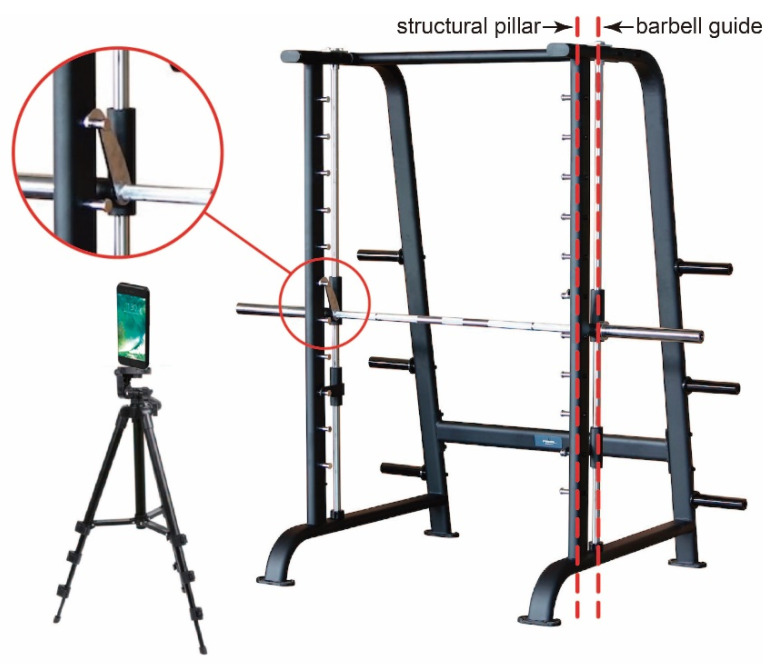
Experimental setup composed of a smartphone with a tripod in front of a multipower machine. Note that the structure and guide planes are parallel (dashed red lines) and that guides need to be clear to allow for a guided movement of the barbell.

The typical scenario of this study consists of an athlete, a coach, or a scientific research team evaluating the performance of training with a multipower machine or an equivalent gym device (Figure 1). In these activities, it is desired to measure the position of the barbell during the exercise to derive the range of motion, velocity, power, and other relevant magnitudes in the field. To acquire this information, several methods and specific instruments are available [21]. This work proposes to use the camera of a smartphone and artificial vision to perform the measurement without the use of specific instruments. Smartphones are widespread devices that pose no incremental cost, with added benefits, like recording video sessions or using the proposed system jointly with other apps to obtain more instant feedback or additional information.

The smartphone camera is the usual monocular rear camera, set in video mode, and held by a tripod facing the multipower machine. It must be located at a distance from the athlete in such a way that the wide dimension of the camera covers a little more than the width of the machine, i.e., approximately 2.2 m for a typical setup. However, the exact position and orientation are not critical because the proposed system includes an algorithm to perform perspective compensation.

The proposed algorithm is briefly described below:

1. The algorithm aims to track the barbell position in the sliding plane using video images.
2. A set of markers will be used to segment the barbell position.
3. Once the barbell position has been located through the markers in the raster images, its position in the real world must be computed.
4. Height is the only information of interest from the real-world position.
5. The physical parameters relevant to this study are extracted from height measurements in each frame and video framerate.

The following steps show the phases of the algorithm, emphasizing the novel solutions employed to solve the tracking problems.

- Section 2.2 is devoted to the fundamentals of the camera model and the proposed set-up geometry.
- Section 2.3 shows a novel method to find the sliding plane from an anterior reference plane in the structure using homography. This step is performed to find the position of the barbell markers in the real world.
- Section 2.4 deals with the automatic detection algorithm of markers from the structure reference plane.
- Section 2.5 is devoted to addressing the automatic detection algorithm of markers from the barbell.
- Section 2.6 details the extraction of the physical variables from the position of the barbell.

### 2.2. Camera Model

**Figure 2:**
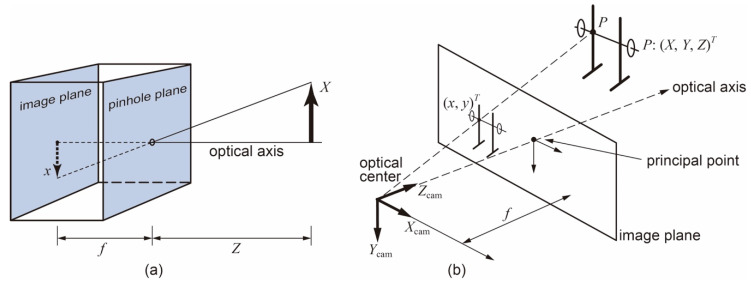
Pinhole camera model used in this study. (**a**) Direct model showing the relationships between inside and outside geometry; (**b**) inverted model defining the geometric relationship between outside coordinates and the corresponding 2D projection onto the image plane.

To describe the image acquisition process, we used the pinhole camera model, projective geometry, and homogeneous coordinates [18,22], as shown in Figure 2a. In this model, the light goes inside the camera through an infinitesimal hole, called the *optical center*, so from each point of the scene, one ray passes through and reaches the rear wall or *image plane*. The relation between the outside and inside geometry is *X*/*Z* = −*x*/*f*, or *x* = −*f·X*/*Z.* The inverted value of *x* with respect to *X* is compensated by the camera software to operate with an upward image, so *x* can be considered as = *f*·*X*/*Z*.

The pinhole camera model can be rearranged into an equivalent scheme by swapping the image and pinhole planes, as depicted in Figure 2b. The point in the pinhole is reinterpreted as the optical center and every ray from the scene is focused on that point. The intersection of the ray and the image plane results in the coordinate of the pixel, i.e., *x* = *f*·*X*/*Z*. This model defines the geometric relationship between a 3D world’s point coordinates and its corresponding 2D projection onto the image plane.

Using homogeneous coordinates and following an appropriate formulation [18,22,23], the relation of a point *P* (*X*, *Y*, *Z*) in the scene and its coordinates (*x*, *y*) on the image plane in pixel units with respect to the upper left corner is:

(1) $$\left(\begin{array}{c} a\\ b\\ c \end{array}\right)\;=\;\left(\begin{array}{ccc} f_{x} & s & c_{x}\\ 0 & f_{y} & c_{y}\\ 0 & 0 & 1 \end{array}\right)\;\left(\begin{array}{c} \begin{array}{c} X\\ Y \end{array}\\ Z \end{array}\right)$$

where *x* = *a*/*c* and *y* = *b*/*c*, *f_x_* and *f_y_* are the focal length in pixel units, *s* is the skew factor of the sensor, and (*c_x_*, *c_y_*) is the position of the principal point with respect to the upper left corner of the sensor. The origin of the 3D coordinates (*X*, *Y*, *Z*) is located in the optical center of the camera.

The cameras of current smartphones have enough quality to assume approximately that *s* = 1, and *f_x_* = *f_y_*. Another important aspect of the camera model is the distortion of the image due to the lens and its position in the smartphone manufacturing process. They can be modeled, respectively, as radial and tangential distortion [23,24]. We performed an analysis of several smartphone cameras with the calibration method developed in [24] and the conclusion is that these distortions are negligible.

The projection phenomenon causes the depth information of the 3D location to be lost. For a given pixel (*x*, *y*), the corresponding point in the scene cannot be unequivocally located as an infinite number of points within the ray between the optical center and the pixel (*x*, *y*) are projected on the same pixel (*x*, *y*). However, if *Z*, or other equivalent information, is available, then (*X*, *Y*) can be obtained. For example, when the points of interest are in a plane of the scene, homography can be applied.

As mentioned at the beginning of Section 2.1, the algorithms of this experiment are applied in current smartphones, which work typically at a 1920 × 1080 pixel resolution and 16:9 aspect ratio, so the vertical length covered is 4 m, and therefore, a spatial resolution of approximately 2 mm (4 m/1920 pixels) is attained. A lower image resolution of 1280 × 720 would also be valid, resolving up to 3 mm. The temporal resolution depends on the number of frames per second. In current smartphones incorporating slow-motion capabilities, 120 or 240 fps give a temporal resolution of 8 or 4 ms. These spatial and temporal resolutions are sufficient for the type of application for which the algorithm is intended.

### 2.3. Homography

**Figure 3:**
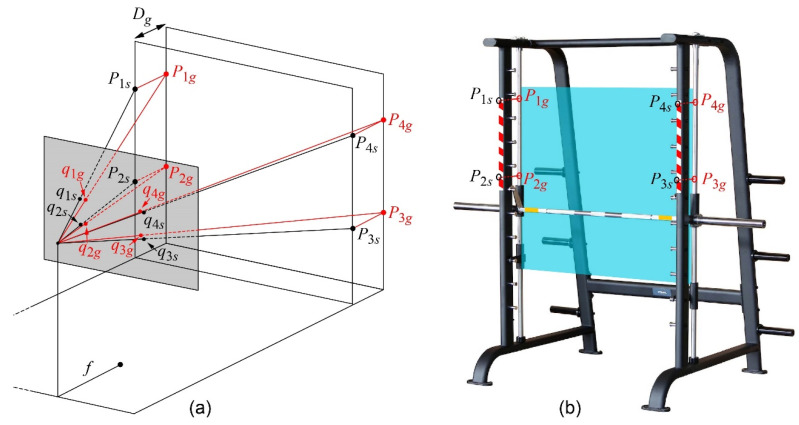
Homography transformations for the measurement of the true magnitude of the barbell movement. (**a**) Model showing the structural *H_s_* and guide *H_g_* homographies with reference points in the structural pillars *P*_1*s*_ to *P*_4*s*_ and guide pillars *P*_1*g*_ to *P*_4*g*_, respectively. (**b**) Location of the reference points in the multipower machine.

The barbell in a multipower machine or similar device is guided by two parallel straight bars, so its movement is developed in a plane, named the barbell plane. The relative position of the image plane and the barbell plane is arbitrary. In the camera, the image of the barbell is projected on the image plane with a perspective projective transformation, but the image pixels can be mapped back to the barbell plane in the 3D world through homography transformation [18]. To this end, four points of the scene in real coordinates and their corresponding pairs in pixel screen coordinates are needed [23,25,26,27]. For this experiment, some known reference marks can be positioned on the multipower machine so that, each time the camera is positioned in front of the athlete and a new measurement is to be started, the system automatically detects the marks and calculates the homography data for that session. However, the marks cannot be located in the barbell plane in these multipower machines since the guide pillars must be free and lubricated for the movement of the barbell (see Figure 1). To solve this problem, the following original procedure is proposed in this work:

1. Some marks are placed on the structural pillars of the machine to define four points: *P_is_*, with *i* = 1, 2, 3, 4, and associated coordinates (*X_is_*, *Y_is_*). Since the structural pillars are always parallel, the four points are in the same plane and therefore homography can be applied. From the quadrilateral formed by these four vertices, six distances are measured: the four sides *D*_12*s*_, *D*_23*s*_, *D*_34*s*_, and *D*_41*s*_, and the two diagonals *D*_13*s*_ and *D*_24*s*_ (Figure 3). Then, the *XYZ* coordinates of the four points in the Cartesian system are determined by the cosine theorem. At this stage, the reference system is placed without loss of generality, with the *XY* axes in the plane defined by the structural columns, so *Z_is_* = 0 for *i* = 1, 2, 3, 4. The origin of coordinates is located at the highest and leftmost reference point *P*_1*s*_.
2. In this type of gym machine, the structural columns and guide columns form two parallel planes (see Figure 1), so the distance between them is also measured as *D_g_*.
3. Estimate homography with reference points located in a practical place; in this experiment, in the two front structural pillars of the machine *P*_1*s*_, *P*_2*s*_, *P*_3*s*_, and *P*_4*s*_.
4. With an appropriate image processing algorithm developed in the following Section 2.4, the four structural reference points are automatically located in the image as *q_is_*(*x_is_*, *y_is_*), *i* = 1, 2, 3, 4., and the homography is computed. It is defined as *H_s_* since the points in the structural pillars are used.
5. The pose and the transformation matrix [28] that relates a generic point *XYZ* of the scene with its pixel *q*(*x*, *y*) are then estimated with *H_s_* and the intrinsic parameters of the camera (Figure 3a). This transformation is called *T_scene2cam_*.
6. Four points located on the guide pillars behind each reference point *P*_1*s*_ to *P*_4*s*_ of the structural plane are defined as *P_ig_* with the following corresponding coordinates: *X_ig_* = *X_is_*, *Y_ig_* = *Y_is_*, and *Z_i_g_* = *D_g_*, *i* = 1, 2, 3, 4, as shown in Figure 3b.
7. With the *T_scene2cam_* transformation, the *q_ig_*(*x*, *y*) coordinates in the image of the points *P_ig_* are computed as (*x_ig_*, *y_ig_*), *i* = 1, 2, 3, 4.
8. With the four corresponding pairs (*X_ig_*, *Y_ig_*), (*x_ig_*, *y_ig_*), *i* = 1, 2, 3, 4, a second homography is calculated, relating the scene plane on the guide and the image plane. This homography is defined as *H_g_* since the points in the guide pillars are used and are intended to map points of the image to points in the *barbell plane* to be able to take measurements in true magnitude.
9. As the camera is located on a tripod and the relative position between camera and machine does not change, the calculated homography *H_g_* is valid in all successive images of the video session. Thus, to calculate the position of the barbell during the athlete’s movement, it is enough to detect the coordinates (*x*, *y*) of the marker located on the barbell in the image, and, through homography *H_g_*, the coordinates (*X_g_*, *Y_g_*) located on the barbell plane are obtained in true magnitude. The six distances of the markers *D*_12*s*_, *D*_23*s*_, *D*_34*s*_, *D*_41*s*_, *D*_13*s*_, and *D*_24*s*_ and the distance to the barbell plane *D_g_* are measured only once and are valid for all training sessions in that machine.

### 2.4. Automatic Detection of Reference Points

The selection of a suitable mark for automatic detection of reference points must consider the following criteria. Firstly, four points in the scene that are located in the same plane are needed for structural homography *H_s_* and their automatic detection has to be robust and precise. Secondly, it is advisable to use a type of signal that a normal user can easily acquire or build. In this study, a self-adhesive warning tape is proposed as a mark signal since it meets both criteria: it can be easily placed in structural pillars and is easy to purchase at a low cost. The placement process of warning tape on the machine is not critical because only two points located in two corners of the left strip and two points in two corners of the right strip will be needed, shown as *P*_1*s*_ to *P*_4*s*_ in Figure 3b. The relative position between the four points can be arbitrary, as long as they are located on the same plane, and this is ensured by the construction of the machine itself, which has two parallel pillars.

**Figure 4:**
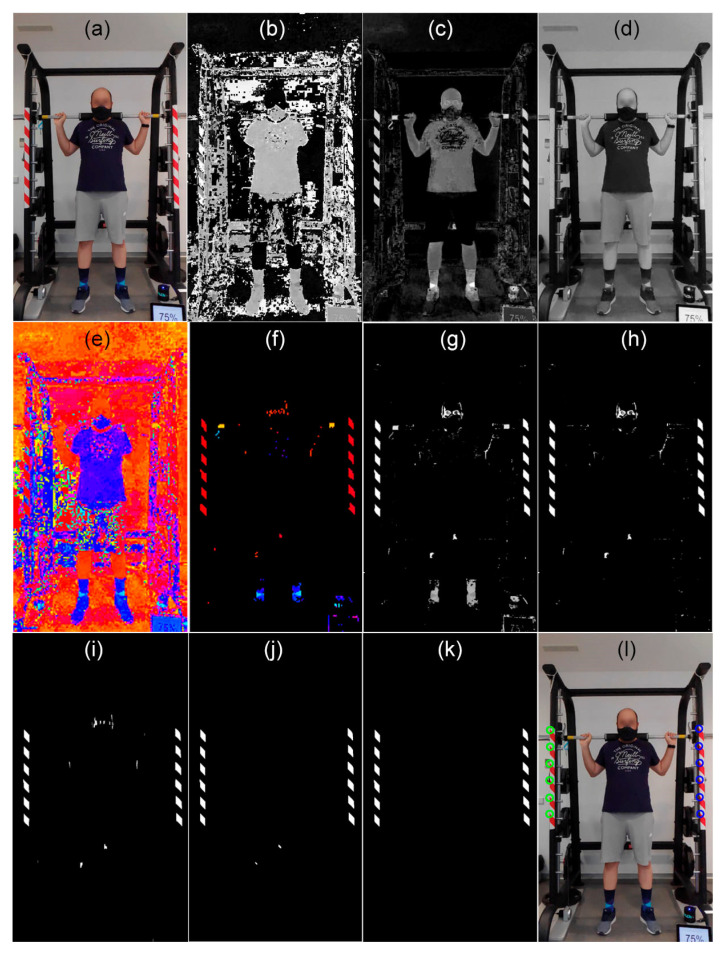
Automatic detection of reference points in the multipower machine. (**a**) Original image. (**b**) Hue, (**c**) saturation, and (**d**) value components of original image. (**e**) Same image as (**b**) but the representation of each value is in colors according to the palette hsv (**f**) Same image as (**e**) but all pixels with saturation lower than 0.5 are set to black. (**g**) *DifHue*, taking as target the hue corresponding to the color red RGB (1, 0, 0). (**h**) Binarized image (**g**). (**i**) Output of the vertical tophat bandpass filter. (**j**) Output of the inclined tophat bandpass filter. (**k**) Filtered image. (**l**) Detected strip points and barbell position.

Although only two points are used on each side, the warning tape provides a sufficiently large area and a substantial number of pixels with content quite different from the global environment of the scene, which allows for robust detection. On the market, the most common tapes available are striped with red–white and black–yellow. In exceptional cases of scenarios where the environment of the machine has content similar in colors or patterns to one type of tape, the user could choose the other option. The following procedure is intended for red–white tape, but a similar process with small changes can be followed for the other color. The process for automatic detection is as follows (see Figure 4):

1. Transform the image from red, green, blue (RGB) to hue, saturation, value (HSV) colorspaces. The hue components of the red color (yellow in the case of black–yellow tape) of the warning tape and a pixel in the image are defined as *hueTarget* and *h*(*x*, *y*), respectively.
2. The image *DifHue*(*x*, *y*) = 1–*circularDif* (*hueTarget*, *h*(*x*, *y*)) is calculated, where the function *circularDif* computes the shortest distance between the hue of the pixel and the target, going clockwise or counterclockwise along the hue scale in a circular way, 1 being the value connected to the value 0.
3. To avoid the influence of pixels with low saturation, all pixels whose value in the *s*(*x*, *y*) component is less than 50% are set to zero in the *DifHue* image. This value of 50% has been selected heuristically and it is not critical.
4. *DifHue* is binarized by selecting those colors that fall within 50% of the range between the target hue and its closest primary or secondary color. The resulting image is called *DifHueBin*.
5. To detect the red-colored polygons, for true (white) regions in the binarized *DifHueBin* image, two size-based mathematical morphology tophat bandpass filters are performed [29,30,31], one with a vertical linear structuring element and another with an inclined linear structuring element at 135 degrees. Each bandpass filter is *BPFilter* = Tophat (*DifHueBin*, *ee*_1_)–Tophat (*DifHueBin*, *ee*_2_), where *ee*_1_ and *ee*_2_ are the structuring element, and their sizes, respectively, twice and half of the expected size of the polygon. Therefore, regions that are larger than twice the expected size or less than half the expected size are eliminated. This image, taken as markers, is reconstructed with the mathematical morphology reconstruction algorithm [32,33].
6. To detect the left warning tape, the left half of the image is selected. The line where the polygons are located is found by adjusting a 1st degree polynomial to the upper left corner of each polygon using the random sample consensus algorithm (RANSAC) and the outliers are eliminated. The upper left corners of the highest and the lowest polygons are selected as reference points for the structural homography *H_s_*. The process is repeated on the right half of the image to detect the tape on the right and obtain its two reference points. Note that this detection process of the reference point occurs only at the beginning of the session and does not need to be updated for each image of the video if the camera is on a tripod.

### 2.5. Automatic Detection of Barbell Markers

**Figure 5:**
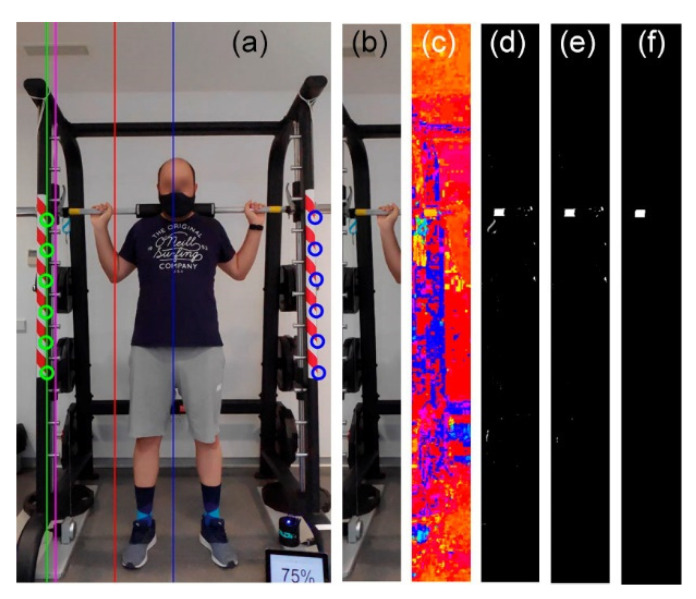
Automatic detection of the left barbell marker. (**a**) Original image with vertical lines showing RANSAC adjustment to the bottom right corners (green); parallel line located to the right (magenta); line dividing the image in half (blue), and a line halfway between magenta and blue (red). (**b**) Subimage selected as the portion between magenta and red. (**c**) Hue component of (**b**). (**d**) 1-*circularDif* to the selected yellow tone. (**e**) Image (**d**) binarized. (**f**) Image (**e**) tophat bandpass filtered.

To improve the robustness of the automatic image processing detection of the barbell movement, a tape of a selected color is glued to the barbell. As depicted in Figure 5a, a yellow piece was used in this experiment, but another color can also be selected. Before the detection process, the area of the image to analyze is limited to minimize the probability of false positives. The bottom right corners of the inlier polygons of the warning tapes are known from the previous reference mark detection process. The green line *L*_1_ in Figure 5a is computed with the RANSAC algorithm. Next, a new parallel line *L*_2_ (magenta), shifted to the right by half the width of the polygons, is estimated.

A vertical strip *S*_1_ from the leftmost point of *L*_2_ (magenta) to the line vertically dividing the image in half (blue) is taken. Finally, the left half of *S*_1_ is used to search the mark, i.e., between the magenta and red lines. All pixels to the left of *L*_2_ are set to zero, resulting in the *ImBarbellStrip* subimage. The process to detect the left barbell mark using this subimage is as follows:

1. The same RGB to HSV conversion process used in the automatic detection of reference points is carried out on the *ImBarbellStrip* image. The calculation of *DifHue* = 1–*circularDif* (*hueTarget*, *h* (*x*, *y*)), the zeroing of the pixels with saturation less than 50%, and the same binarization performed for the detection of the pillars are also computed.
2. Next, the tophat bandpass filter is again applied with a structuring element twice and half the expected size of the mark, this time with a vertical and horizontal structuring element.
3. To detect the right barbell mark, the three preceding steps are repeated, but for the right part of the image. Depending on the scene, noise, glowing areas, etc., there may be no, one, or more candidates detected on each side.
4. With the candidates on the left and right sides, the pair with the closest horizontal coordinate between them is selected. If a candidate is missing, it is filled in by interpolation with the images of the previous and subsequent moments.

The entire detection process described in Section 2.3 and steps 1 to 5 of Section 2.4 are carried out with the images supplied by the camera without making any kind of perspective correction. The conversion of coordinates with the *H_g_* homography is carried out only on the coordinates of the upper left corner of the marks detected on the barbell, which is a process with a computational cost much less than performing the perspective correction on all the pixels of the image.

### 2.6. Data Analysis

The different kinetic and kinematic variables relevant to VBT were calculated, taking into consideration only the time when the barbell was moved by the athlete in the concentric or lifting phase [11]. The main source of information was the instantaneous position of the barbell through time measured with the tracking capability of the algorithm. The total range of displacement of the barbell was then computed as the difference between the position taken the instant before the beginning of the ascent of the barbell and the position when the barbell ceased movement. The instantaneous vertical velocity was calculated as the change in position over time within the range of displacement. The instantaneous acceleration was then computed by differentiating the velocity data with respect to time. To calculate the force exerted by an athlete, the displacement of the center of mass is considered to be similar to the displacement of the barbell [34]. Therefore, the instantaneous force *F* applied to the system is

(2) $$F\;=\;\left(m_{a}+m_{l}\right)\times \left(a_{b}+g\right)$$

where *m_a_* is the body mass of the athlete, *m_l_* is the mass of the external load, *a_b_* is the acceleration of the barbell, and *g* is the acceleration due to gravity. Instantaneous power is then calculated as the product of force and barbell velocity. Mean values of these variables were computed considering the time interval required to complete the concentric range of motion of each repetition.

### 2.7. Instrument Validation

Twenty recreationally active male athletes (age 23.6 ± 4.1 y, height 181.9 ± 5.8 cm, and body mass 85.8 ± 11.5 kg) visited the laboratory twice, separated by 2 weeks. On the first visit, athletes performed a standardized 1RM (one-repetition maximum) test [35] of back squats, consisting of two trials with a 3 min rest between repetitions, starting at 20 kg and progressively increasing in 15 kg increments until the mean velocity was lower than 0.7 m/s. Later, the attained load was incremented with smaller weights between 2.5 and 5 kg until the heaviest load each athlete was able to lift was considered the maximum load or 1RM. During the second visit, the mean velocity values were measured to the following percentages of 1RM: 75%, 85%, 90%, and 95%, reflecting moderate to high velocities in the back squat to test the video system under the most unfavorable conditions for tracking. Athletes performed two repetitions of each percentage with a 3 min rest between repetitions. They were instructed to abstain from drinking caffeinated beverages or alcohol for 24 hours before both testing occasions. The study was carried out in accordance with the guidelines of the ethical principles of the Declaration of Helsinki. All subjects provided informed written consent before the beginning of this study, which was approved by the University Institutional Review Board (IRB No. UA-2019-01-19).

The back squat exercise was performed on a multipower machine (ProStrength Multipower Professional, Pro-Gym, Barcelona, Spain), measuring 150 × 126 × 227 cm. The barbell instantaneous position was simultaneously monitored by the proposed method (video) and a commercial linear position transducer or LPT serving as a criterion method [8,36]. The video method consisted of a smartphone (Pocophone F1, Xiaomi, Pekin, China) placed on a tripod positioned 2.2 m in front of the machine (Figure 1) to track the entire range of movement of barbell markers and machine reference points (Figure 3). Video recordings were taken with the settings of current smartphones (definition of 1920 × 1080 pixels and frame rate of 240 fps). The LPT method consisted of the linear position transducer Chronojump (Chronojump Boscosystem, Barcelona, Spain), which comprises an optical rotatory encoder with a retractable cable that is attached to the barbell using a collar provided by the manufacturer. The LPT device was connected to a laptop running the manufacturer’s software (Chronojump v2.0, Chronojump, Barcelona, Spain) with a sampling frequency of 1000 Hz. The device was located under the barbell on the vertical displacement axis and attached to a magnetic weight plate on the floor.

### 2.8. Statistical Analysis

Descriptive statistics are presented as mean ± SD, and 95% confidence intervals (95% CIs). The reliability of the video method was tested using two-way random single measurements (absolute agreement) intraclass correlation coefficient (ICC) (2,1), and Cronbach’s *α* [37]. ICC values were interpreted as poor (<0.5), moderate (0.5–0.75), good (0.75–0.9), and excellent (>0.9) reliability [38]. Furthermore, paired-sample *t*-tests and mean differences with 95% CIs, which represent uncertainty in the true value, were used to analyze the outcome differences between video and LPT systems. The smallest worthwhile change (SWC), measuring the minimum improvement likely to have a practical impact, was calculated via standardization as 20% of the between-subjects standard deviation [3]. The usefulness of the proposed instrument was evaluated by comparing the SWC and the typical error of measurement (SEM) [39]: the ability of the video system to detect changes is assessed with the ratio of the SWC to SEM, interpreted as good (>1), satisfactory (1), and marginal (<1) [40]. Bland–Altman plots were also used to explore the agreement between the two instruments [41], which show mean outcome pairs against their difference between values to identify any random error and proportional bias with a bivariate Pearson’s product-moment correlation coefficient of *r*^2^>0.1 [1]. Finally, the validity of the two instruments was calculated with the bivariate Pearson’s product-moment correlation coefficient (*r*) with 95% confidence intervals (CIs), using the following thresholds: trivial (<0.1), small (0.1–0.3), moderate (0.3–0.5), high (0.5–0.7), very high (0.7–0.9), and practically perfect (>0.9) [42]. The standard error of estimate (SEE) was computed in raw units and standardized, evaluated via *r* to allow estimation of confidence limits [43], and interpreted using half the thresholds of the modified Cohen’s scale: trivial (<0.1), small (0.1–0.3), moderate (0.3–0.6), large (0.6–1.0), very large (1.0–2.0), and extremely large (>2.0) [42]. All statistical analyses were computed with IBM SPSS v. 22 (IBM Corp, Armonk, NY) and an available spreadsheet for validity [44].

## 3. Results

### 3.1. Comparison between Instruments

**Figure 6:**
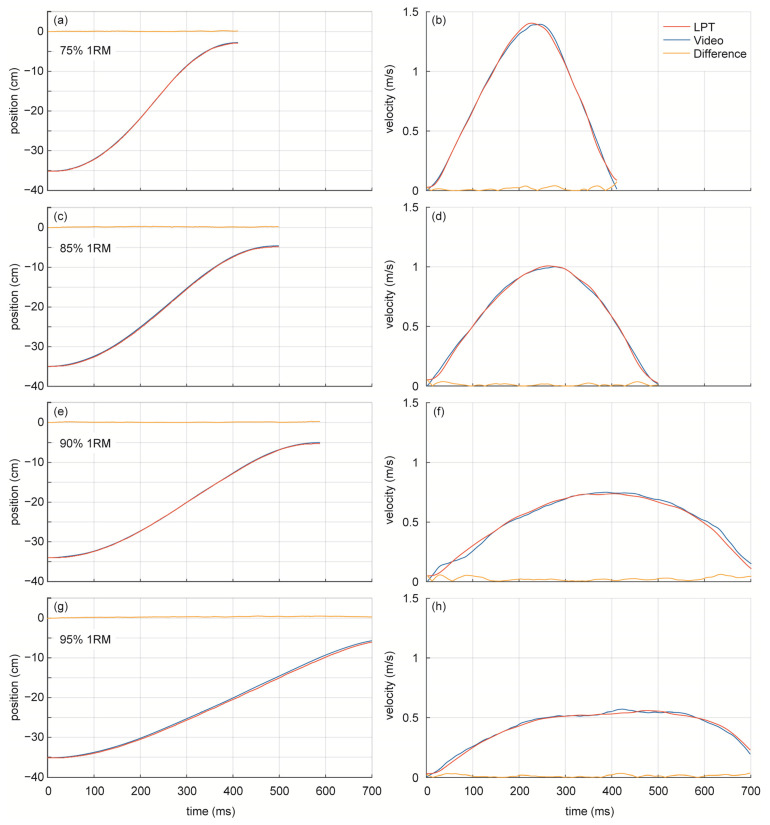
Position–time and velocity–time curves for one repetition of linear position transducer (LPT) system (blue line), video system (red line), and difference between systems (orange line); (**a**,**b**) 75% 1RM; (**c**,**d**) 85% 1RM; (**e**,**f**) 90% 1RM; (**g**,**h**) 95% 1RM.

The instantaneous position tracked by the video system and the LPT system, together with derived instantaneous velocity for the four 1RM percentages, are shown in Figure 6 for one repetition. Since the primary source of information is the measurement of the position of the barbell over time, the goodness of fit will depend on the consistent outcomes of both systems. The first column of Figure 6 shows practically identical measures of position for the video system (blue line) and LPT system (red line) for all 1RM percentages. The point-by-point difference (orange line) depicts almost null values across the ranges of 75% 1RM (−3.2 to −35.1 cm), 85% 1RM (−4.9 to −35.0 cm), and 90% 1RM (−5.0 to −34.1 cm) and differences below 1 cm for the range of 95% 1RM (−5.9 to −35.1 cm).

With regard to instantaneous velocity, the second column of Figure 6 depicts the typical shape of velocity–time curves as 1RM percentages increase. For low percentages (75% RM), meaning low additional weights, velocity reaches its maximum value across all 1RM percentages (1.42 m/s) for both systems. When weights increase and, therefore, 1RM percentages, maximum velocity tends to decrease: 1.0 m/s for 85% 1RM, 0.73 m/s for 90% 1RM, and 0.56 m/s for 95% 1RM. In all cases, the computed instantaneous velocity is consistent across the video system and LPT system, meaning that the outcomes of both systems are equivalent.

**Figure 7:**
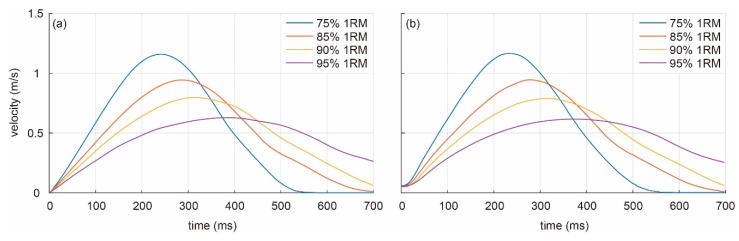
Velocity–time curves averaged across all subjects for (**a**) LPT system and (**b**) video system. Blue line: 75% 1RM; red line: 85% 1RM; yellow line: 90% 1RM; purple line: 90% 1RM.

Velocity is the most important outcome in VBT practice, so the velocity–time curves averaged across all athletes were computed for both systems, and each 1RM percentage (Figure 7). The shape of each curve changes with 1RM percentage for both systems: as additional weight increases, the velocity of the barbell decreases (1.16, 0.95, 0.81, and 0.63 m/s for 75%, 85%, 90%, and 95% 1RM, respectively) and the time to reach maximum velocity increases (242, 288, 318, and 392 ms for 75%, 85%, 90%, and 95% 1RM, respectively). The similarity between velocity–time curves can be seen by comparing Figure 7a,b).

**Figure 8:**
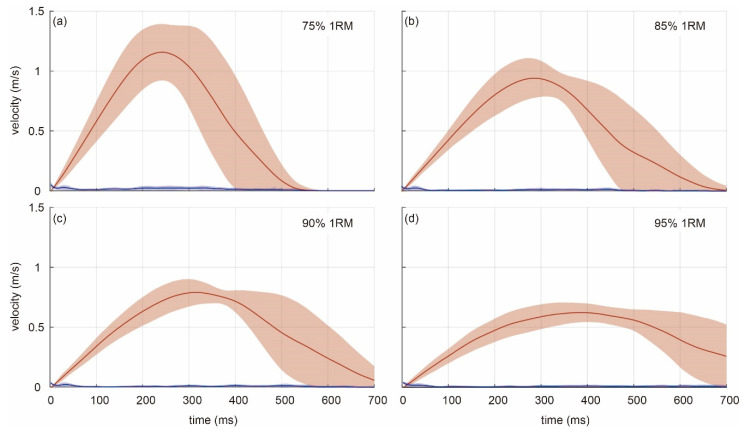
Velocity–time curves averaged across all subjects for the video system at (**a**) 75% 1RM, (**b**) 85% 1RM, (**c**) 80% 1RM, and (**d**) 95% 1RM. Velocity (red line) and absolute difference between LPT and video systems (blue line) shown with standard deviations (shaded area).

The velocity–time curves can also be analyzed with a graphical representation of standard deviations, measuring the variance of the measure, together with the instantaneous velocity averaged across all subjects. Figure 8 shows the output of the proposed video system (red) and the absolute difference between systems (blue), all as mean (lines) and standard deviations (shaded areas). The variability for lower weights and higher velocities (75% 1RM) is larger than that of higher weights and lower velocities (95% 1RM). As with the previous representations, both systems provided very similar outcomes, judging from the low absolute difference between the video system and LPT system (lower than 0.02 m/s).

### 3.2. Instrument Validation

The agreement between the proposed video system and the LPT system was studied through the 160 lifts executed by 20 athletes. For every repetition, the instantaneous variables of velocity, force, and power were averaged over the time of the concentric phase of the execution (lifting phase), as is commonly carried out in VBT [5,6]. The resulting descriptive statistics showed ranges (mean ± SD) of 33.8 ± 4.9 cm for the video system and 33.4 ± 4.8 cm for the LPT system. Regarding mean velocity, the video system provided 0.58 ± 0.12 m/s whereas the LPT system led to 0.57 ± 0.12 m/s. The computed mean force exerted by athletes resulted in 1691 ± 85 N for the video system and 1675 ± 89 N for the LPT system. Finally, mean power resulted in 973 ± 163 W for the video system and 943 ± 164 W for the LPT system. All paired comparisons between the proposed video system and the LPT system resulted in statistical significance (*p* < 0.01), meaning that the outcomes for both systems are significantly similar to one another.

The intraclass correlation coefficient for the video system showed excellent agreement for all variables: range, ICC = 0.996; velocity, ICC = 0.988; force, ICC = 0.978; power ICC = 0.979, as shown in Table 1. Likewise, Cronbach’s α coefficients near unity demonstrated excellent reliability. The proposed video system revealed negligible underestimation of range (−0.35 ± 0.24 cm), velocity (−0.016 ± 0.093 m/s), force (−15.94 ± 8.98 N), and power (−30.26 ± 15.47 W), compared to the LPT system (*p* < 0.01).

The usefulness of the video system was evaluated using the smallest worthwhile change (SWC), the minimum practically meaningful change in a variable due to real enhancements over the noise of the measure. The SWC resulted in 0.97 cm, 0.02 m/s, 17.75 N, and 32.98 W for range, velocity, force, and power, respectively.

**Figure 9:**
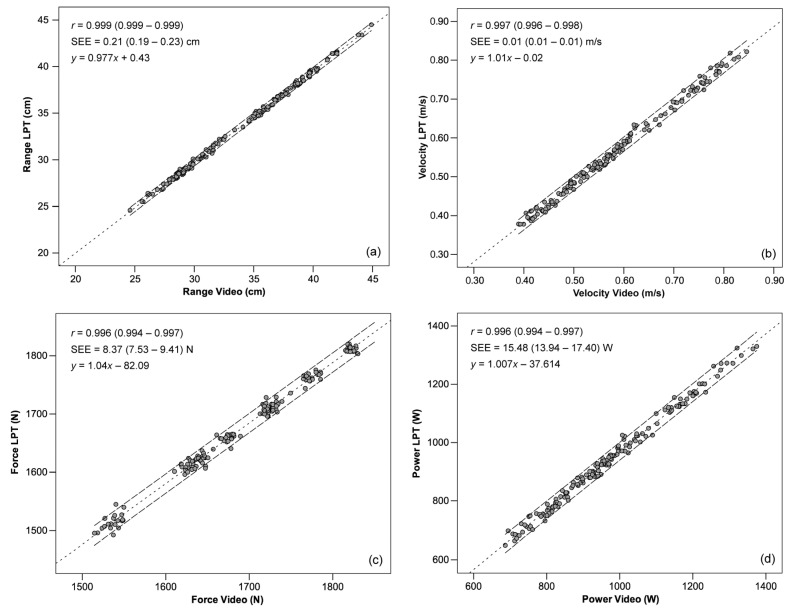
Bland–Altman plots for the measurements of LPT and video systems. Solid central line represents mean between instruments (systematic bias); upper and lower dashed lines show mean ± 1.96 SD (random error); dotted line shows linear regression (proportional bias). (**a**) Range: regression *y* = –0.02*x*+0.41 cm, *r*^2^ = 0.22; (**b**) velocity: regression *y* = 0.01*x*–0.02 m/s, *r*^2^ = 0.03; (**c**) force: regression *y* = 0.04*x*–88.13 N, *r*^2^ = 0.17; (**d**) power: regression *y* = 0.01*x*–41.71 W, *r*^2^ = 0.02.

Bland–Altman plots showed high levels of agreement between the video system and the LPT system because most of the paired measurements fell within the dashed lines in Figure 9, representing the 95% limits of agreement given by ±1.96·SD of the differences. Furthermore, very low mean systematic bias ± random errors were observed for range: –0.35 ± 0.47 cm, velocity −0.016 ± 0.018 m/s, force −15.94 ± 17.6 N, and power −30.26 ± 30.34 W, being all *p* < 0.01. The difference between the two systems was steady with increasing values of velocity (*r*^2^ = 0.03), force (*r*^2^ = 0.17), and power (*r*^2^ = 0.02). As a result of the homoscedasticity of the errors, no association between the magnitude of the errors and the mean value of these variables was expected (*r*^2^ < 0.1) [45,46]. The only variable showing proportional bias is the range (*r*^2^ = 0.22), although the association is weak.

**Figure 10:**
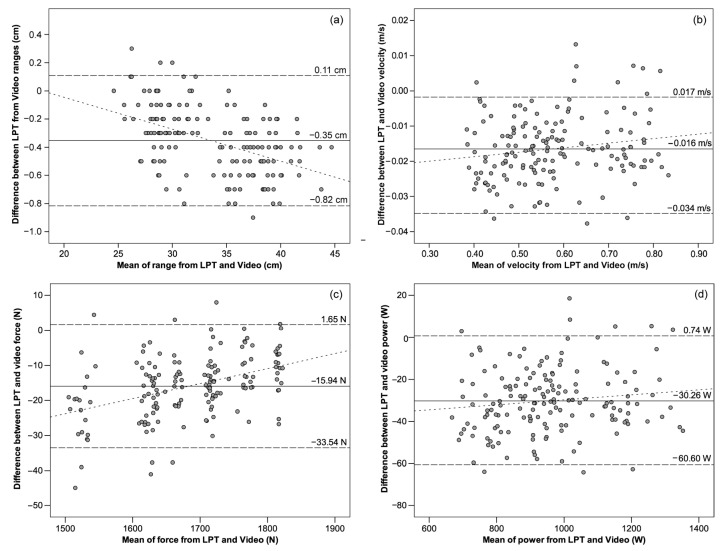
Relationship between measurements derived from LPT and video systems. Dotted line represents linear regression; upper and lower dashed lines show 95% confidence intervals. (**a**) Range; (**b**) velocity; (**c**) force; (**d**) power. Pearson’s product-moment correlation coefficient (*r*) and standard error of estimate (SEE) shown with 95% confidence intervals between brackets; *p* < 0.01.

The bivariate Pearson’s product-moment correlation coefficient showed practically perfect association (*r* = 0.999 for range, *r* = 0.997 for velocity, and *r* = 0.996 for force and power, being all *p* < 0.01) between the video system and LPT system, as shown in Figure 10. Likewise, the regression lines provided very accurate predictions, as given by the low standard error of estimates for range (0.21 cm), velocity (0.01 m/s), force (8.37 N), and power (15.48 W). According to the effect size, these errors are considered trivial.

## 4. Discussion

The aim of this paper was to present and validate a new video-based instrument that provides unattended, real-time measures of barbell velocity with a smartphone high-speed camera. To that end, the proposed image processing algorithm allows for the automatic tracking of barbell markers and detection of reference points of a multipower machine to perform autocalibration in a contactless way. To the best knowledge of the authors, this is the first video-based instrument providing real-time barbell velocity outcomes without prior manual measurements of reference points in the scene [10,17,20,47], and without human errors due to manual video frame by frame inspection [10,17,20] or manipulation of the smartphone chronometer [47].

In recent years, smartphone-based instruments have received much scientific attention for the assessment of physical activity and sports training, given their ubiquity in the population and the ability to install specialized applications [48]. In the field of VBT, as an efficient force training, the velocity of weight displacement can be regarded as the most important parameter to monitor and prescribe individually tailored training programs [4]. Velocity can be derived either from instantaneous displacement differentiation using position-tracking instruments, with LPT being the most popular instrument [11,49], or from acceleration integration using inertial measurement units [50]. The first type of instrument relies on accurate measurement of the dynamic position of the object of interest, namely, the barbell in force training. In a video-based method like the one proposed in this study, the primary sources of information are time and two-dimensional space and, therefore, both temporal and spatial resolution must be considered. Our system has been tested with a high-speed camera setting of 240 Hz (fps or frame per second), which is present in most current smartphones, which allows for a temporal resolution of 1/240 = 4.2 ms. However, a sampling frequency in measuring resistance training exercises of above 25 Hz is adequate to record raw velocity data and compute derived parameters, such as force and power [51], so video recordings could have been performed at lower frame rates. Considering peak velocities of ~1–1.4 m/s for low weights [5], the distance between barbell markers between consecutive frames is 0.83–1.17 cm for 120 fps or 0.42–0.58 cm for 240 fps. Contrastingly, when the information retrieval is performed by human digitizing, including human errors due to observation, low video frame rates may pose severe limitations when estimating velocity outcomes at high velocity barbell displacement (>0.80 m/s) [52]. The automatic tracking of the proposed system avoids such human errors, so lower frame rates could be used, as with LPT encoders in [51]. With regard to the spatial resolution, our system has demonstrated that the recording resolution of current smartphones of 1920 × 1080 pixels allows for a 2.1 mm resolution for a vertical length covered of 4 m. This resolution is higher than 1280 × 720 in *My Lift* [10,17,20], that manually selects the start and end frames of the concentric phase of the execution (lifting phase). For the same length covered, the spatial resolution decreases to 3.7 mm, compared to the proposed video system.

The automatic detection of barbell markers in uncontrolled environments like gyms or fitness centers, where smartphone position and light conditions may change, was solved by using distinct colors or patterns so that the computer vision algorithm can discriminate robustly [53]. In our system, two strips of self-adhesive warning tape with an oblique red–white pattern were used as reference points to define the multipower machine geometry, and a simple small piece of colored tape is used to track the barbell. A recently released optical instrument to measure barbell velocity bypasses the detection and segmentation problem using infrared reflective markers on a scale that the user must locate in the plane of movement before the execution and a set of additional markers in the barbell [15,36]. Contrastingly, in our proposal, the plane of movement is autocalibrated with materials available to everyone.

The position–time and velocity–time curves for one repetition of the video system showed very close agreement of the instantaneous outcomes with respect to the validated LPT [8,36]. The variance observed in the peak values of velocity–time curves averaged across all subjects for the video system is larger at lower weight percentages (~±0.3 m/s for 75% 1RM) than at higher percentages (~±0.1 m/s for 95% 1RM). The latter may be due to dissimilar degrees of biological variability of athletes in the lift executions depending on the amount of displaced weight. However, concerning the accuracy of the video system, the small blue shaded area surrounding the mean of the difference between systems (blue in Figure 8) suggests no significant variance in the difference because both systems detect very similar outputs.

The validation of the proposed video system has been performed by comparison with another system considered as a criterion. The reliability of the video system was excellent for all variables due to ICC values greater than 0.9 and narrow confidence intervals. Our results are in accordance with previous studies of manual video-based smartphone apps for back squat exercises (*My Lift*: ICC = 0.981 [17], ICC = 0.972 [10], for velocity peak values). Similarly, a Cronbach’s α coefficient of 0.99 indicated excellent consistency for range, velocity, force, and power outcomes.

The level of agreement between the two systems measuring the same variables was also tested using Bland–Altman plots [41]. In this study, all variables demonstrated a very low systematic bias of −0.35 cm for range, −0.016 m/s for velocity, −15.94 N for force, and −30.26 W for power. Hence, the proposed video system displays negligible underestimation with respect to the LPT system. Analogous bias was observed in the *My Lift* app for peak velocity (−0.005 m/s [17] and −0.001 m/s [20]), underestimating outcomes with respect to LPT systems. The lower systematic values of these studies with respect to ours could be due to the different type of kinematic variables under test (peak vs. mean velocity). The same app tested for mean velocity gave similar results to the proposed video system (0.01 m/s [11]). For the study of a smartphone app measuring mean velocity values across a series using timekeeping [10], the systematic bias is even larger and negative (–0.022 m/s), although all errors are negligible in comparison to typical velocity ranges. Likewise, the random errors given by the narrow limits of agreement observed for all the study variables (range: ± 0.47 cm, velocity: ± 0.018 m/s, force: ± 17.6 N, and power: ± 30.34 W) suggested that the video system shows less error variance than *My Lift* for peak (± 0.04 m/s [17], ± 0.28 m/s [20]) and mean velocity values (±0.05 m/s [11]), and other apps (± 0.034 m/s [47]). The Pearson’s product-moment correlation and the regression line of the measured average values and the differences between systems in a Bland–Altman plot can reveal if the systematic error is steady and independent of the sample of measured values [1]. Our results show no association between the systematic mean value and the magnitude of the random errors of mean velocity, as *r*^2^ < 0.1, in accordance with other studies of *My Lift* with peak velocity values (*r*^2^ = 0.016 [17]) and other apps with mean values (*r*^2^ = 0.01 [47]). Since the random error is low and stable irrespective of the velocity range measured, the proposed video system is able to detect typical small changes in velocity needed to train and monitor high-performance athletes [46].

The concurrent validity was tested using the bivariate Pearson’s product-moment correlation coefficient between paired outcomes. The proposed video system provided valid measures of range for all variables, with practically perfect associations between systems (*r* = 0.99), in accordance with *My Lift* assessed with mean velocity values (*r* = 0.99 [11]). However, this coefficient dropped slightly when peak values were measured with the same app (*r* = 0.965, 0.902, 0.963, [10,17,20], respectively) or other apps with manual timekeeping (*r* = 0.948 [47]). Similarly, a low standard error of estimates (SEE) of the regression lines for all variables demonstrated very accurate predictions and low variability in the outcomes (range: 0.21 cm, velocity: 0.01 m/s, force: 8.37 N, power: 15.48 W). Our results showed lower SEE values than *My Lift* both for peak (0.04 m/s [17], 0.124 m/s [20]) and mean velocity (0.096 [10]).

The usefulness of the video system can be calculated as the ratio between the smallest worthwhile change (SWC), representing the minimum change in performance to be considered meaningful, and the standard error of measurement (SEM), assessing the uncertainty of the measure. Our results show that the minimum enhancement likely to demonstrate a practical impact was 0.97 cm for range, 0.02 m/s for velocity, 17.75 N for force, and 32.98 W for power. These SWC values were computed as a fraction of the between-subjects standard deviations [54] to set a threshold below which measures have no practical significance. In contrast, the video system produced a very low uncertainty of 0.31 cm, 0.01 m/s, 13.17 N, and 23.89 W for range, velocity, force, and power, respectively. Similar uncertainty has been reported for peak velocity values of *My Lift* (0.02 m/s [10]). The SWC to SEM ratio provides essential information on the usefulness of the proposed video system. When SWC is greater than SEM, the instrument is able to detect changes over the noise of the measure. For the video system, the SWC/SEM ratio is greater than unity for all variables, so the signal-to-noise ratio of the practical measurement of range, velocity, force, and power allows for a meaningful assessment of changes in sports performance. For the case of mean velocity, a ratio of 2.0 implies that the uncertainty of the measure in the video system of 0.01 m/s is half the minimum improvement likely to be substantial, 0.02 m/s, so the proposed video system is sensitive enough to monitor variations in velocity over the uncertainty of the measuring process [39].

In this study, the back squat with a multipower machine was used to demonstrate the validity and reliability of the proposed video system due to the popularity among VBT practitioners. Future studies may test the instrument for the tracking of moving weights, such as in bench press exercises [55], where higher velocity values can present a challenge for current high-speed cameras, or free exercises like the snatch, where reference points for autocalibration cannot be placed on a static geometric structure, but on the barbell.

## 5. Conclusions

This study has presented a new instrument for measuring barbell velocity with a smartphone high-speed camera using image-processing algorithms. The proposed system can detect reference points of a multipower machine to perform autocalibration, and track barbell markers, analyze the kinematics of the movement, and give real-time VBT performance metrics. The entire measurement is an automatic, contactless, and unattended process for the user, hence avoiding errors due to human frame-to-frame video digitizing. The proposed video system can be regarded as a trustworthy instrument that provides valid and reliable measures of velocity and derived parameters similar to dedicated devices.

## Figures and Tables

**Table 1:** Reliability of video and LPT systems.

	Range	Velocity
ICC (2,1)	0.996 (0.881−0.999)	0.988 (0.542−0.997)
Cronbach’s α	0.999	0.999
Mean Difference	−0.35 * (−0.39–−0.31) cm	−0.016 * (−0.018–−0.015) m/s
SWC	0.97 (0.87–1.09) cm	0.02 (0.02–0.03) m/s
SEM	0.31 cm	0.010 m/s
SWC/SEM Ratio	3.16	2.00
SEE	0.21 (0.19–0.23) cm	0.01 (0.01–0.01) m/s
Standardized SEE	0.04 (0.04–0.05)	0.08 (0.07–0.09)
SEE Effect Size	Trivial	Trivial
	Force	Power
ICC (2,1)	0.978 (0.370−0.994)	0.979 (0.296−0.995)
Cronbach’s α	0.997	0.998
Mean Difference	−15.94 * (−17.25–−14.45) N	−30.26 * (−32.78–−27.78) W
SWC	17.75 (15.99–19.96) N	32.98 (29.70–37.08) W
SEM	13.17 N	23.89 W
SWC/SEM Ratio	1.35	1.38
SEE	8.37 (7.53–9.41) N	15.48 (13.94–17.40) W
Standardized SEE	0.09 (0.08–0.11)	0.09 (0.08–0.11)
SEE Effect Size	Trivial	Trivial

Data expressed with 95% confidence intervals where appropriate; ICC: Intra-class correlation coefficient; SWC: Smallest worthwhile change, SEM: Standard error of measurement; SEE: Standard error of estimate; * *p* < 0.01.

## Data Availability

The data presented in this study are available on reasonable request from the corresponding author.
